# Structural Insight into the *Clostridium difficile* Ethanolamine Utilisation Microcompartment

**DOI:** 10.1371/journal.pone.0048360

**Published:** 2012-10-29

**Authors:** Alison C. Pitts, Laura R. Tuck, Alexandra Faulds-Pain, Richard J. Lewis, Jon Marles-Wright

**Affiliations:** 1 Institute for Cell and Molecular Biosciences, Newcastle University, Newcastle upon Tyne, United Kingdom; 2 Department of Pathogen Molecular Biology, London School of Hygiene and Tropical Medicine, London, United Kingdom; 3 Institute of Structural and Molecular Biology, School of Biological Sciences, University of Edinburgh, Edinburgh, United Kingdom; MRC National Institute for Medical Research, United Kingdom

## Abstract

Bacterial microcompartments form a protective proteinaceous barrier around metabolic enzymes that process unstable or toxic chemical intermediates. The genome of the virulent, multidrug-resistant *Clostridium difficile* 630 strain contains an operon, *eut*, encoding a bacterial microcompartment with genes for the breakdown of ethanolamine and its utilisation as a source of reduced nitrogen and carbon. The *C. difficile eut* operon displays regulatory genetic elements and protein encoding regions in common with homologous loci found in the genomes of other bacteria, including the enteric pathogens *Salmonella enterica* and *Enterococcus faecalis*. The crystal structures of two microcompartment shell proteins, CD1908 and CD1918, and an uncharacterised protein with potential enzymatic activity, CD1925, were determined by X-ray crystallography. CD1908 and CD1918 display the same protein fold, though the order of secondary structure elements is permuted in CD1908 and this protein displays an N-terminal β-strand extension. These proteins form hexamers with molecules related by crystallographic and non-crystallographic symmetry. The structure of CD1925 has a cupin β-barrel fold and a putative active site that is distinct from the metal-ion dependent catalytic cupins. Thin-section transmission electron microscopy of *Escherichia coli* over-expressing *eut* proteins indicates that CD1918 is capable of self-association into arrays, suggesting an organisational role for CD1918 in the formation of this microcompartment. The work presented provides the basis for further study of the architecture and function of the *C. difficile eut* microcompartment, its role in metabolism and the wider consequences of intestinal colonisation and virulence in this pathogen.

## Introduction

The human gut is a complex and highly competitive ecosystem that is populated by many different species of bacteria, each adopting different strategies to survive within the niches they inhabit [1]. Pathogenic species are usually out-competed by the commensal species that make up the healthy gut microbiota [2], [3], but they often make use of toxins directed towards other bacterial species [4], or the host [5] to enable them to colonise environments that would otherwise be occupied by competitors, or to create new niches through changes to their host organism. *Salmonella enterica* and *Escherichia coli* species are common causes of gastroenteritis and diarrheal illness in the healthy population [6], [7], while *Clostridium difficile* is a major cause of hospital acquired diarrhoea and has significant risks of morbidity and mortality in the elderly and immune compromised patients [8], [9]. With an ageing population that is becoming increasingly reliant on hospital care, there is much interest in understanding the molecular basis of the metabolism of *C. difficile* and its role in intestinal colonisation and virulence.

Nutritional stress induces the expression of the *C. difficile* toxins, which act on host cells and induce an inflammatory response [10], [11]. As a consequence of the cellular damage and ensuing inflammation caused by these toxins large quantities of phospholipids, particularly the abundant phosphatidylethanolamine, are liberated from the cell membranes of host epithelial cells and other bacteria [12], [13]. Phosphatidylethanolamine is broken down readily by bacterial phosphodiesterases into glycerol and ethanolamine [14], and a number of enteric pathogens, including *S. enterica*, *Enterococcus faecalis* and some species of *Clostridia* can use ethanolamine as a sole source of nitrogen and carbon [15]–[18]. Indeed, an association between ethanolamine metabolism and virulence in *S. enterica* is emerging [19], [20].

The breakdown of ethanolamine is carried out by a two-subunit adenosylcobalamin (AdoCbl) cofactor-dependent ethanolamine ammonia lyase protein complex, which is encoded by the genes *eutB* and *eutC* [21]. These genes are usually associated with a number of accessory proteins that activate the AdoCbl cofactor and allow the efficient conversion of the acetaldehyde produced by this enzyme into acetyl-CoA, which can then be used in various metabolic processes, such as the TCA cycle in those bacteria capable of aerobic respiration, lipid biosynthesis, or for substrate level phosphorylation to generate ATP [22]. The genes associated with the ethanolamine ammonia lyase vary widely between species; some bacteria are only able to utilise ethanolamine as source of reduced nitrogen, as they possess only the lyase genes, while others possess a long operon encoding regulatory elements and a number of proteins that are homologous to carboxysome shell proteins [23]–[27]. These metabolic compartments allow the efficient utilisation of various carbon sources and are termed bacterial microcompartments (BMCs) [28]. The sequestration of ethanolamine metabolism within a BMC is thought to protect the cell from the acetaldehyde produced as an intermediate in its breakdown and to prevent the loss of this volatile compound and its carbon from the cell [29].

The biochemistry of the ethanolamine utilisation has been extensively studied in *Salmonella* species [15], [21], [30]–[32] However, there remain a number of questions about the roles of some of the enzymes associated with the ethanolamine ammonia lyase [15], [18], [32], [33]. Work on the structure of this BMC is limited to X-ray crystal structures of a number of the shell proteins from the *E. coli eut* operon [34]. To understand the function of the ethanolamine utilisation BMC, it is necessary to understand its architecture, including the features that are unique to this particular class of BMC and those that are conserved across carboxysomes and other BMCs. Knowledge of the structure of the ethanolamine utilisation BMC will form the basis for exploring its wider impact on bacterial metabolism and virulence. It is also apparent that BMCs have the potential for exploitation as protein containers for nano-technology and synthetic biology [35]–[37].

**Figure 1:**
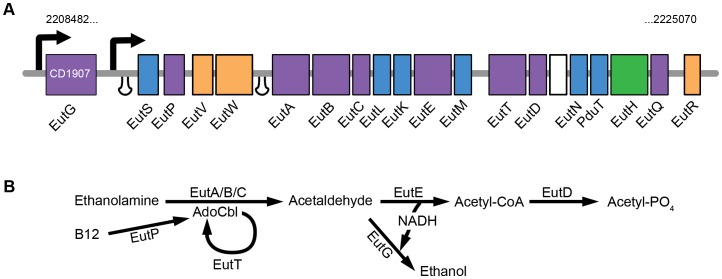
Schematic of the *C. difficile eut* operon and enzymatic pathway. A. The genomic position of the operon is labelled above the encoded genes, which are colour coded according to their proposed function; Purple boxes represent genes with putative enzymatic activities; orange boxes, genes encoding regulatory proteins; blue boxes, genes with homology to bacterial microcompartment proteins; green box, gene encoding a trans-membrane transporter; and the white box, gene with unknown function. Intergenic regions are shown as large gaps between identified coding sequences, putative promoter elements and transcriptional terminator loops are shown as arrows and loops respectively. **B**. Proposed pathway and roles of specific enzymes in ethanolamine utilisation by *C. difficile*.

The ethanolamine utilisation (*eut*) operon of the virulent multi-drug resistant *Clostridium difficile* strain 630 encodes an ethanolamine ammonia lyase and associated accessory proteins; enzymes required for the utilisation of the organic carbon liberated by the lyase; regulatory proteins and six proteins with homology to the carboxysome shell proteins [38] (Fig. 1, Table 1 and Supporting Information S1for detailed description of the operon). To understand the structure and function of the *C. difficile eut* bacterial microcompartment we have determined the crystal structures of two proteins with homology to BMC shell proteins, CD1908 and CD1918, and show that they are well conserved between species. We have also determined the atomic structure of CD1925, a member of the EutQ family of cupin barrels. A putative active site is identified that is metal ion independent and unique in the wider cupin family. The cytoplasm of *E. coli* cells over-expressing these proteins revealed arrays formed by CD1918 *in vivo*, which are analogous to the arrays seen for the PduA protein from the propanediol utilisation microcompartment of *Citrobacter freundii* and EtuA from the ethanol utilisation microcompartment from *Citrobacter kluyveri*.

**Table 1:** *C. difficile eut* operon organisation.

Locus tag	Start	Stop	Protein homologue	Comment
CD1907	2208482	2209612	EutG	Alcohol dehydrogenase
CD1908	2209853	2210203	EutS	BMC protein
CD1909	2210211	2210645	EutP	GTPase
CD1910	2210754	2211329	EutV	Response regulator
CD1911	2211322	2212731	EutW	Sensor histidine kinase
CD1912	2212915	2214348	EutA	Reactivating factor
CD1912	2214366	2215730	EutB	Ethanolamine ammonia lyase
CD1914	2215743	2216624	EutC	Ethanolamine ammonia lyase
CD1915	2216644	2217297	EutL	BMC protein
CD1916	2217308	2218009	EutK	BMC protein
CD1917	2218012	2219481	EutE	Aldehyde dehydrogenase
CD1918	2219567	2219854	EutM	BMC protein
CD1919	2219981	2220742	EutT	Cobalamin adenosyltransferase
CD1920	2220755	2221384	EutD	Phosphotransacetylase
CD1921	2221426	2222151		Unknown function
CD1922	2222164	2222436	EutN	CcmL pentameric shell protein
CD1923	2222429	2222980	PduT	BMC protein, Iron sulphur cluster
CD1924	2222992	2224083	EutH	Ethanolamine transporter
CD1925	2224085	2224558	EutQ	Cupin
CD1926	2224678	2225070	EutR	AraC regulator

The stop and start sites of genes with overlapping reading frames are underlined. CD1926 is encoded on the complementary strand.

## Results

### Structural analysis of *C. difficile eut* operon proteins

To understand the function and macromolecular organisation of the individual proteins within the *C. difficile eut* operon, a number of *eut* genes were cloned into pET28b for overexpression and subsequent structural analysis by X-ray crystallography and transmission electron microscopy (TEM). The genes encoding CD1908, CD1918 and CD1925 were expressed to high levels both as C-terminal His_6_ tagged and untagged variants; the tagged variants of these proteins were subjected to crystallisation and their structures determined by X-ray crystallography, while the native proteins were subjected to TEM to assess the formation of higher-order structures *in vivo*.

### Crystal structure of CD1908, the *C. difficile* EutS homologue

**Figure 2:**
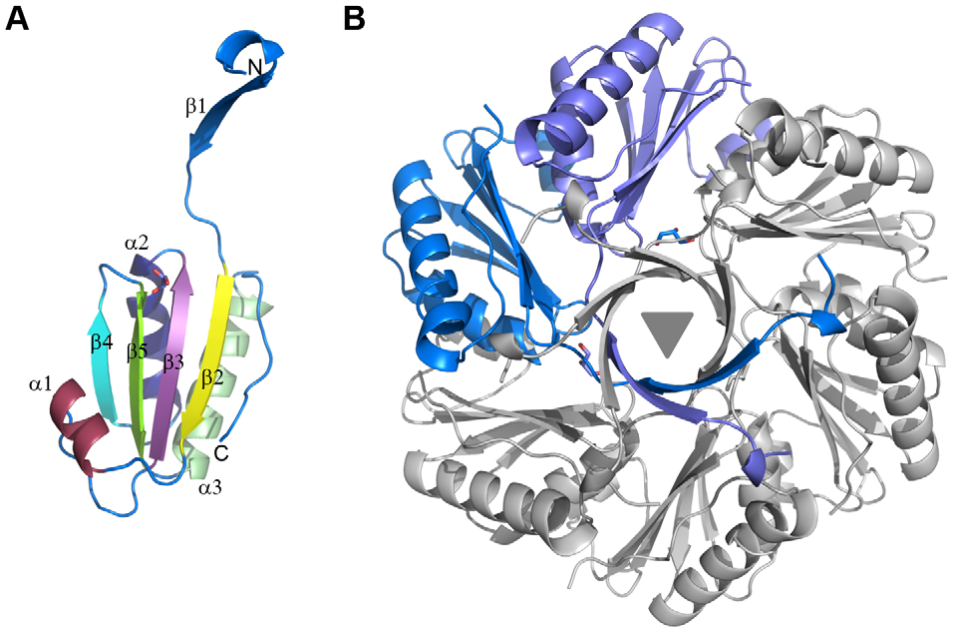
X-ray crystal structure of CD1908. A. Cartoon representation of the CD1908 monomer with secondary structure elements coloured and labelled from N- to C-termini. **B**. Hexameric arrangement of CD1908 showing the asymmetric unit contents in blue and symmetry related molecules in grey, with the three-fold symmetry axis generating this arrangement shown as a grey triangle.

The structure of CD1908 was determined by molecular replacement to 1.51 Å resolution. Two molecules are present in the asymmetric unit, comprising the complete native protein sequence, less the N-terminal methionine, with a single amino acid visible from the C-terminal His_6_ tag in chain A. CD1908 displays a permuted BMC domain fold [39], with a four-stranded anti-parallel β-sheet flanked by two α-helices on one face and one helix on the other with an N-terminal β-strand extension (Fig. 2A). The protein forms a hexamer with pairs of molecules related by the crystallographic 3-fold axis (Fig. 2B). The two chains in the asymmetric unit are virtually identical, with an rms Cα deviation of 0.4 Å over 115 aligned residues. A glycerol molecule from the crystallisation condition was found associated with each chain. The structure was refined with anisotropic B-factors and the final refined model had an R_cryst_ of 0.140 and R_free_ of 0.180 (see Table 2 for data collection and refinement statistics).

**Table 2:** Crystal parameters and data collection statistics for *C. difficile* Eut proteins.

		EutS	EutM	EutQ_(17–157)_
Data collection				
	Space group	H 3	I 1 2 1	C 1 2 1
	Wavelength (Å)	0.9793	0.98040	0.8266
	Cell dimensions			
	a, b, c (Å)	123.2, 123.2, 38.7	69.4, 46.4, 76.4	82.33, 66.57, 58.95
	α,β,γ (°)	90, 90, 120	90, 91.56, 90	90, 117.54, 90
	Resolution	61.6–1.51(1.59–1.51)	38.2–1.62(1.71–1.62)	43.15–1.00(1.05–1.00)
	No. observations	131,851 (19,163)	110,091 (16,241)	343,892 (43,410)
	No. unique observations	34,286 (5,001)	30,458 (4416)	148,309 (21947)
	Multiplicity	3.8 (3.8)	3.6 (3.7)	2.3 (2.0)
	Completeness (%)	99.7 (99.9)	98.3 (97.9)	97.9 (99.4)
	Mean I/sigma I	12.4 (2.5)	12 (3.5)	11.5 (2.4)
	R_sym_	0.058 (0.495)	0.063 (0.356)	0.058 (0.356)
Molecular Replacement Model				
		3CGI [40]	2EWH [69]	2PYT
Refinement				
	Number of reflections	34,282	30,452	14,8298
	R_cryst_	0.140	0.166	0.136
	R_free_	0.180	0.196	0.147
	Number of atoms (no-H)	1,930	2,062	2,648
rmsd				
	bond lengths (Å)	0.016	0.015	0.014
	bond angles (°)	1.68	1.55	1.60
Ramachandran Plot				
	Favoured (%)	99.20	100	98.97
	Allowed (%)	0.80	0.00	1.03
B-factors				
	Wilson B (Å^2^)	18.6	18.4	7.6
	Main chain (Å^2^)	16.2	9.9	11.7
	Side chain (Å^2^)	23.4	17.9	15.4
	Water (Å^2^)	30.8	30.2	24.7
	Ligand (Å^2^)	33.1	45.8	n/a
PDBID		4AXI	4AXJ	4AXO

Values in parentheses are for the highest resolution shell.

**Figure 3:**
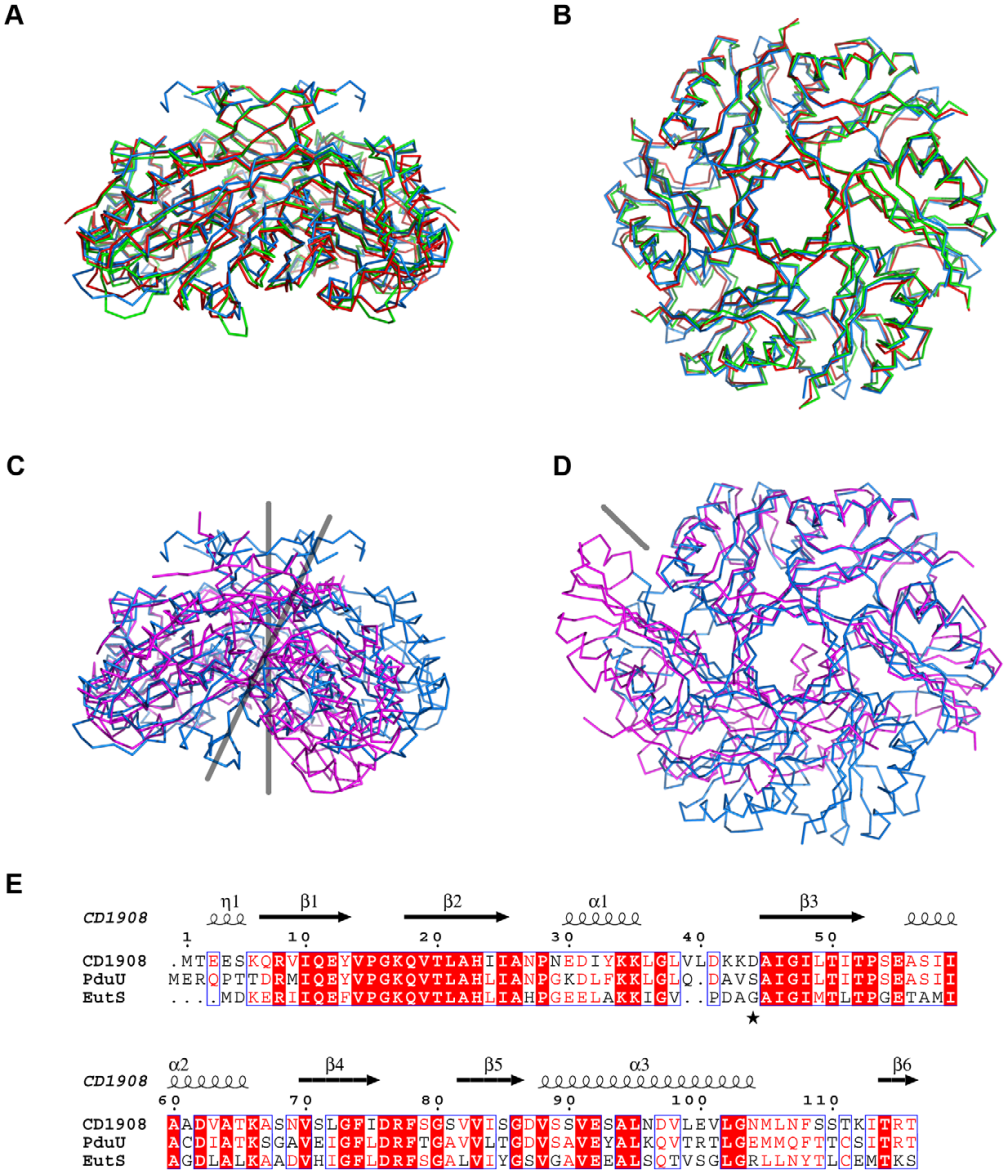
Alignment of CD1908 and its homologues. **A**. Ribbon view of a structural alignment of CD1908 (blue), PduU (green) and EutS_(G39V)_ (red). **B**. Orthogonal view of A, viewed down the hexameric axis. **C**. Alignment of CD1908 with the bent EutS hexamer, the orientation of the central axes are shown with grey lines. **D**. Orthogonal view of C, viewed down the multimeric symmetry axis. Rather than being a symmetric hexamer, EutS displays a split arrangement of two trimers.

A single CD1908 monomer superimposes on PduU from *S. enterica* (PDBID: 3CGI) [40] and EutS from *E. coli* (PDBID: 3IA0) [34] with root mean square Cα deviations of 0.9 Å and 1.1 Å, respectively, over 111 aligned residues. The CD1908 hexamer adopts a conformation almost identical to both PduU and to the EutS_(G39V)_ mutant (Fig. 3A/B); it superimposes on PduU with an rms Cα deviation of 0.97 Å over 662 residues with 65% sequence identity, while EutS_(G69V)_ superimposes with an rms Cα deviation of 1.05 Å over 657 residues with 53% sequence identity. The flat CD1908 hexamer is in contrast to the bent and displaced arrangement seen for the wild type EutS protein (Fig. 3C/D). In wild type EutS, the hexamer is split into two trimers that are offset by roughly 17 Å and the central axis is bent by roughly 40° from the conformation seen in EutS_(G39V)_, PduU and CD1908. The region of the protein containing the equivalent residues to Gly39 of EutS is an external loop and is poorly conserved between the three proteins. In CD1908 this residue is an aspartic acid and PduU has a serine in this position (Fig. 3E). The structural basis for why changes to this residue cause a large rearrangement of the EutS multimer is not clear, and Tanaka *et al* [34] offer no hypothesis as to why changes at this surface would affect the formation of the hexamer in such a way.

Both CD1908 and PduU have longer extensions to their N-terminal β-barrel than EutS, although at only three and four residues longer respectively, they do not constitute a significant increase in length (Fig. 3E). This region is visible in the structure of CD1908 and loops back over the core of the protein to form a short 3_10_ helix that contacts the main body of the protein.

### Crystal structure of CD1918, the *C. difficile* EutM homologue

**Figure 4:**
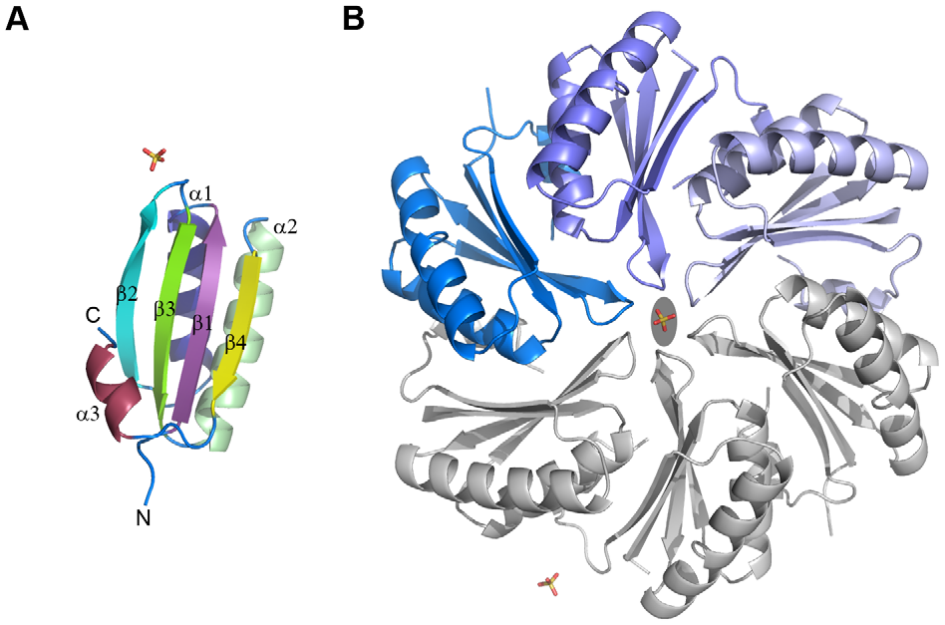
Crystal structure of CD1918. **A.** Cartoon representation of the CD1918 monomer with secondary structure elements coloured and labelled from N- to C-termini. **B.** Hexameric arrangement of CD1918 showing the asymmetric unit contents in blue and symmetry related molecules in grey; the two-fold crystallographic symmetry axis that generates this arrangement is shown as a grey ellipse.

The structure of CD1918 was determined by molecular replacement to 1.62 Å. Three protein chains were present in the asymmetric unit, with each chain visible in electron density to residue ninety of the polymer. The chains superimpose with an average rms Cα deviation of 0.5 Å over the whole protein chain. The structure was refined with isotropic B-factors and the final refined model has an R_cryst_ of 0.166 and R_free_ of 0.196 (see Table 2 for data collection and refinement statistics). CD1918 displays the canonical BMC fold with a four-stranded anti-parallel β-sheet flanked by α-helices (Fig. 4A), and forms a hexamer with molecules related by a crystallographic 2-fold axis (Fig. 4B). Two sulphate ions are present at crystallographic symmetry axes. CD1918 shares 77% sequence identity with PduA from *S. enterica* (PDBID:3NGK) [41], and 72% with EutM from *E. coli* (PDBID: 3MPW) [42]. It superimposes on these proteins with root mean square Cα deviations of 0.5 and 0.7 Å respectively over 86 aligned residues, forming identical hexameric arrangements to these proteins.

### Functional implications for the structures of CD1908 and CD1918

**Figure 5:**
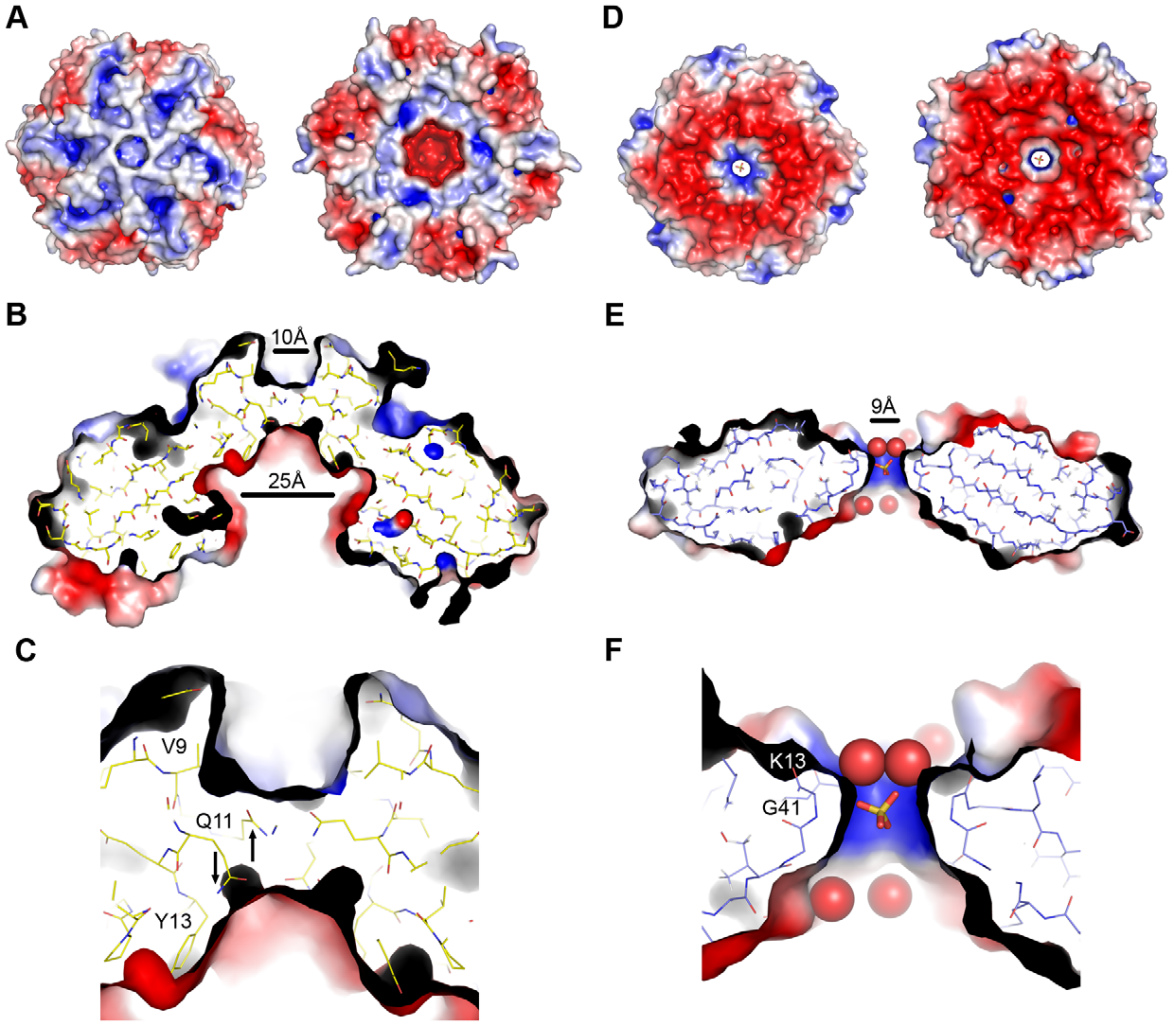
Electrostatic surfaces and characteristics of CD1908 and CD1918 hexamers. **A**. The electrostatic potential of the solvent accessible surface of CD1908 was calculated using the default parameters in PDB2PQR [67] and APBS [68] and mapped to the molecular surface in PyMol and displayed over a cartoon representation of the molecule. Blue indicates regions of positive potential (> +5 kT/e) and red indicates negative potential (< −5 kT/e). Both faces of the structure are shown. **B**. Cross-section view of the CD1908 pore with the electrostatic surface and stick view of the protein molecule shown. The diameter of the pore at the top and bottom of the hexamer is shown. **C**. Enlarged view of the top of the pore from panel D showing the positions of the conformationally flexible Gln11 residue with arrows indicating the two conformations seen in the crystal structure. **D**. Electrostatic potential of the surface of the CD1918 hexamer displayed as described for panel A. **E**. Cross-section of the CD1918 hexamer displayed in the same manner as panel A, in this molecule the pore is open and contains a sulphate ion with ordered solvent molecules visible in the pore. **F**. Enlarged view of the top of the pore showing the peptide nitrogen of Gly41 and side-chain of Lys13, which form the boundary of this opening.

The microcompartment shell must recruit and encapsulate those enzymes required for its function and allow the passage of substrates and products through the shell. The structures of BMC proteins give insight into their roles in organising the structure of microcompartments and their function. Both CD1908 and CD1918 form homo-hexamers in solution that presumably function as the building blocks of the *C. difficile* ethanolamine utilisation microcompartment shell. CD1908 does not have an open pore at the centre of the hexamer [41], [42] (Fig. 5A), instead the central region of the oligomer interface is formed by the N-terminal β-barrel extension and this is blocked by Glu11 (Fig 5B/C). This residue adopts multiple conformations in both chains in the asymmetric unit; due to stereochemical constraints these were modelled in alternating up and down conformations for the two chains in the asymmetric unit. The N-terminal face of CD1908 has distinct patterns of positive charge on the surface (Fig. 5A), with a shallow depression at the centre of the oligomer that is approximately 10 Å in diameter and blocked by Glu11. The opposite face of the protein has a distinct conical cavity that is 25 Å wide at its widest point and has Glu11 at its apex (Fig. 5B/C). This cavity is lined with aromatic residues, including Tyr13 and Phe78. The apex of this cavity is predominantly negatively charged, while the aromatic residues give the base of the cone a hydrophobic character. These features are in accord with those seen for its *S. enterica* homologue PduU [40] and the EutS_(G39V)_ structure. The native EutS is also blocked by an equivalent glutamic acid at the apex of the cone, but because the hexamer is skewed, these residues adopt different conformations to accommodate the different organisation of the monomers in the structure.

The oligomer of CD1918 has much flatter faces than CD1908 and it has an open pore at its centre (Fig. 5D). The faces of CD1918 are primarily negatively charged, with positive patches around the sides of the hexamer and a positively charged pore. This pore is 9 Å in diameter and its boundary is demarcated by a loop between β-strands 1 and 2, with the amide nitrogen of Gly41 and the side-chain of Lys41 (Fig. 5E/F) forming the boundary of the pore. Electron density consistent with a sulphate molecule, a component of the crystallisation condition, was built within the pore, and there are a series of water molecules above and below the pore. The structure of EutM also contains a bound sulphate ion in this pore. The presence of a sulphate ion within this pore, and its narrow width, suggest that small polar, or charged, molecules would be the most probable candidate ligands for transport through the BMC shell by this protein. This protein may be a channel for water, or a conduit for the transport of the ethanolamine substrate, or for the exit of the acetyl-phosphate produced by the phosphotransacetylase encoded by CD1920.

### Structure of CD1925, a EutQ family cupin

**Figure 6:**
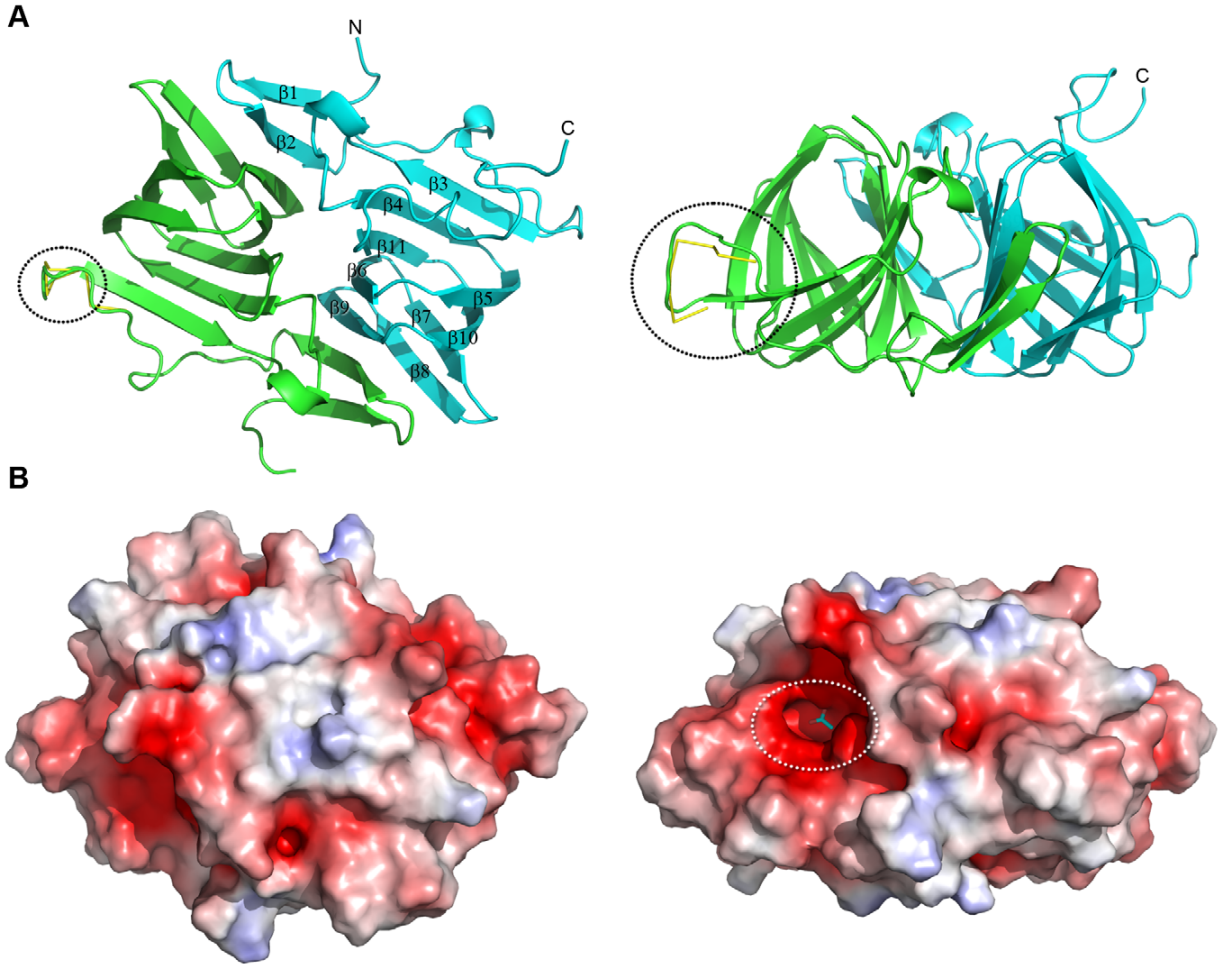
Structure of CD1925. **A**. Cartoon view of the asymmetric unit contents in the CD1925 crystal. A dimer is formed between the two cupin-barrels present in the asymmetric unit, coloured green and cyan, with secondary structure elements labelled. The loop between residues 56 and 64 in chain A that adopts two conformations is highlighted with a black circle. Orthogonal views are shown at left and right with the molecule rotated into the plane of the figure. **B**. Electrostatic potential of CD1925 mapped onto the molecular surface of the protein, calculated and displayed as for Fig. 3 with positive potential shown in blue ( > + 5 kT/e) and negative potential shown in red (< −5 kT/e). A surface cleft is visible in the view at left, highlighted by the dotted white oval. An acetate ion has been modelled into this cleft to illustrate its scale.

The full length CD1925 protein did not produce diffracting crystals, therefore a series of N-terminal truncations were generated to remove this potentially unstructured portion of the protein. The crystal structure of a CD1925 variant, truncated at the N-terminus by 16 amino acids (CD1925_(17–157)_), was solved by molecular replacement and determined to 1 Å resolution. The structure was refined with anisotropic B-factors and the final refined model had an R_work_ of 0.136 and an R_free_ of 0.147 (see Table 2 for data collection and refinement statistics). Two chains were present in the asymmetric unit (Fig. 6A), with residues 28–153 visible in chain A and 29–157 visible in chain B, with the majority of the C-terminal His_6_ tag also visible in the latter chain. The protein belongs to the EutQ family of cupins and has the distinct β-barrel fold associated with these proteins. One face of the barrel is formed by β-strands 3, 4, 11, 6 and 9 and the other by strands 5, 10, 7 and 8; strands 1 and 2 at the N-terminus of the protein fall outside of the conserved cupin core. The two chains in the asymmetric unit form a dimer, with a total interface area of 1740 Å^2^ out of 9285 Å^2^ total surface area for each monomer. This dimer interface is formed between one face of the β-barrel of each monomer, with an interaction between β9 of one monomer and β2 of the partner chain, thus extending the β-sheet across the two molecules. The two chains superimpose with an rms Cα deviation of 0.52 Å over 124 aligned residues. The loop between residues 56 and 64 in chain A adopts two distinct conformers modelled with 0.55 and 0.45 occupancy (Fig. 6A black circle).

**Figure 7:**
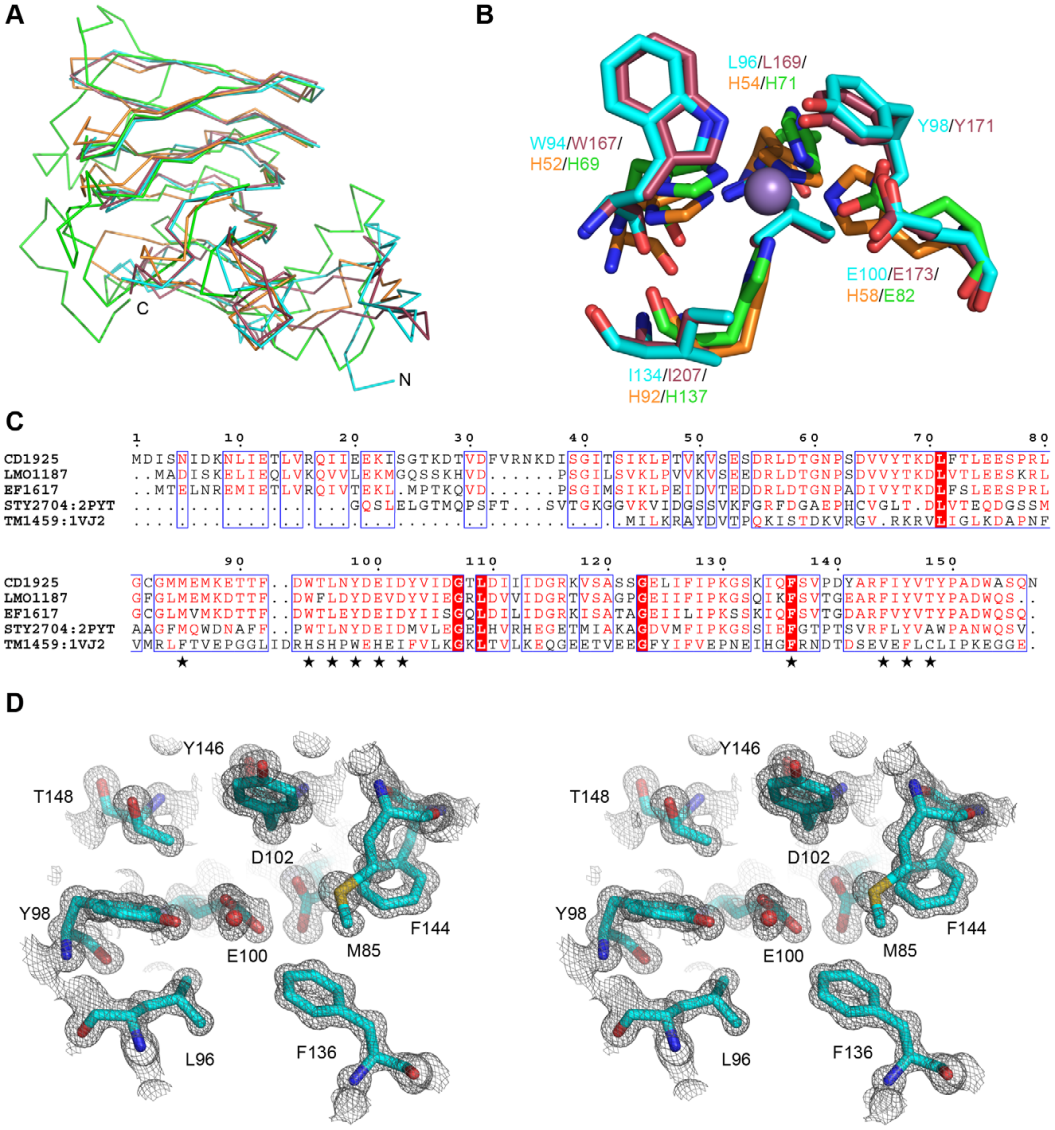
Potential active site of CD1925. **A**. Ribbon view of the structure of CD1925 with homologous structures superimposed. CD1925 is shown in cyan, the structures of the homologous cupins, EutQ from *Salmonella typhimurium* (PDBID: 2PYT, red) and metal binding cupin from *Thermotoga maritima* (PDBID: 1VJ2, orange) and *Bacillus subtilis* (PDBID: 2Y0O, green), are shown superimposed on CD1925. Despite low sequence homology within the cupin family, the core architecture of the fold is almost identical. **B**. Metal coordinating residues from 1VJ2 and 2Y0O are shown with their equivalent residues from CD1925 and 2PYT, molecule and residue colours are the same as for A. Where the metal binding cupins have histidine residues, the EutQ homologues possess aromatic and hydrophobic residues and share a conserved glutamic acid residue with the catalytic cupin 2Y0O. **C**. Sequence alignment of CD1925 and homologues from *Listeria monocytogenes* (LMO1187), *Enterococcus faecalis* (EF1617), 2PYT and 1VJ2. Residues lining the proposed active site are well conserved among EutQ type proteins, shown with stars, and distinct from the equivalent positions in the metal binding cupins represented by 1VJ2. **D**. Stereo view of the proposed active site of CD1925. Residues lining the cleft shown in Fig. 6B are displayed as sticks with their final 2mFo-dFc electron density shown as a grey mesh and contoured at 1.5σ.

The surface of CD1925 does not display the distinctive charge distribution patterns seen for the oligomeric BMC proteins CD1908 and CD1918; instead its surface has irregular patches of positive and negative electrostatic potential (Fig. 6B). A shallow, negatively charged cleft is visible on the surface of each protein chain within the cupin β-barrel (Fig. 6B white oval), this cleft corresponds to the region that coordinates divalent cations and acts as the active site in the metal binding cupins, such as those with sugar isomerase activity [43] (Fig 7A/B). CD1925 shares 30% sequence identity with EutQ from *S. typhimurium* (PDBID: 2PYT, unpublished structural genomics output) and superimposes with an rms Cα deviation of 1.25 Å over 116 amino acids. Despite low sequence identity with metal binding cupins, such as those from *Thermotoga maritima* (PDBID: 1VJ2) [44] (14%), and *Bacillus subtilis* (2Y0O) [45] (12%), these structures superimpose with rms Cα deviations of 1.88 and 1.74 Å respectively, over 98 residues in both cases. The conservation of the cupin core is evident in these structures, with the majority of structural differences occurring at the termini of the proteins and loops that extend from the core (Fig. 7A).

In contrast to the metal-binding catalytic cupins, CD1925 and the EutQ family, do not possess the histidine residues that are responsible for metal coordination in the oxidoreductase [46] and epimerase [47] classes of cupins. In the place of the histidine residues are aromatic (Trp94) and hydrophobic residues (Leu 96, Ile134) and in the fourth position a glutamic acid residue (Glu100) (Fig. 7B/C), which is also present in the cupins with epimerase activity. The glutamic acid (Glu100) is within hydrogen bonding distance of an aspartic acid residue (Asp102), which may act to alter its pKa. Because of strong sequence conservation seen in this area for the EutQ family and the fact that this region is solvent accessible and it corresponds with the position of the catalytic site of other cupins, this region may act to bind ligands, or act as a catalytic centre (Fig. 7C, starred residues).

### Higher order structures formed by *C. difficile* Eut proteins

**Figure 8:**
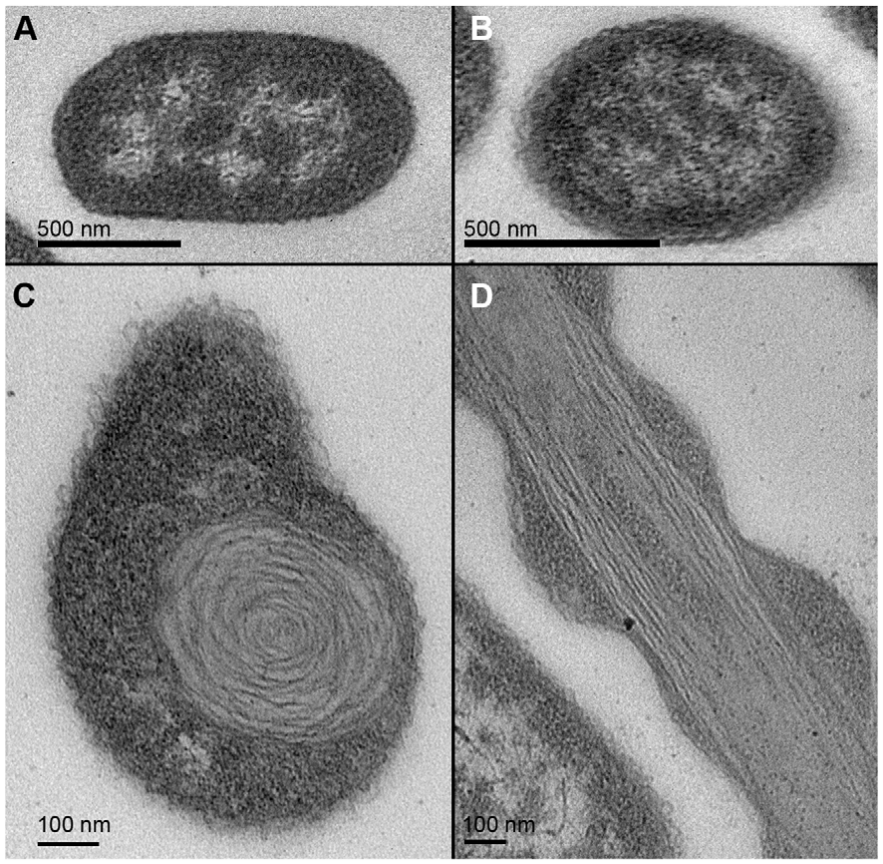
Thin-section transmission electron micrographs *of E. coli* cells over-expressing C. difficile Eut proteins. **A**. All cells display a normal cellular morphology in *E. coli* producing CD1908, image taken at a microscope magnification of 34,000 x. **B**. *E. coli* cells over producing CD1925 also display normal cellular morphology, image taken at a microscope magnification of 34,000 x. **C**. Transverse views of *E. coli* cells over-expressing CD1918, around 90% of cells display significant internal morphological changes, 10 nm wide lamellar structures are visible as rose-shaped arrangements, image taken at 92,000 x magnification. **D**. Longitudinal views of *E. coli* cells overexpressing CD1918 show that the structures formed extend throughout the cytoplasm in bunches, where they appear to interfere with cell division in around 90% of cells seen in this view, image taken at 64,000 x magnification.

The interactions between the proteins encoded in BMC loci produce an enclosed microcompartment that has a shell made solely of protein [39]. This shell envelops the enzymes required for the metabolic function of the BMC and allows the passage of substrates and products, while preventing the escape of metabolic intermediates. Transmission electron microscopy of thin sections of *E. coli* cells overexpressing carboxysome proteins from *Halothiobacillus neapolitanus* [35], *Citrobacter freundii* propanediol utilisation BMC proteins [48], *Clostridium kluyveri* ethanol utilisation BMC [49] and *S. enterica* ethanolamine utilisation proteins [37], have revealed the presence of higher-order protein structures. To understand the possible roles that the proteins in this study may play in determining the organisation of the *C. difficile eut* BMC shell, *E. coli* cells individually transformed with the expression plasmids for the untagged BMC shell proteins CD1908 and CD1918 and the enzyme CD1925 were analysed for the formation of protein arrays. Untagged proteins were used to avoid any potential artefacts arising from the interaction of recombinant tags added to the proteins and to ensure the native protein structures were conserved. *E. coli* strains containing the plasmids encoding the full length untagged proteins were grown to exponential phase and induced with 1 mM IPTG for three hours. Thin sections of fixed cells were imaged by TEM and assessed for the presence of any higher-order structures. Neither the uninduced controls, nor CD1908 and CD1925 produced any morphological changes to, or within, the cells (Fig. 8A/B). Our results and those seen for the *C. kluyveri* PduU protein [48] are in contrast to those obtained by Chaudhary *et al* [37], who show that the *S. enterica* EutS protein is able to form enclosed compartments when over-expressed in *E. coli*. It is notable that the crystal structures of CD1908 and *S. enterica* PduU do not have the bent arrangement of the native *E. coli* EutS that gives this protein the ability to form enclosed structures [37]. The EutS_(G39V)_ mutant, which has the same arrangement as CD1908 and PduU, is also unable to form enclosed polyhedra. Why CD1908 should display a greater degree of sequence and structural conservation to the *S. enterica* PduU protein than EutS is unknown; given the former is part of a BMC with a distinct metabolic function, this raises questions as to the role that these proteins may play in the formation of the BMC shell and its function. Given that *C. kluyveri* can support a functional ethanol utilisation BMC with an ethanol utilisation operon that encodes only two shell proteins [49], it is conceivable that with six encoded shell proteins in the *C. difficile eut* operon, there is scope for these proteins to perform different roles in the formation of a functional BMC shell.

**Figure 9:**
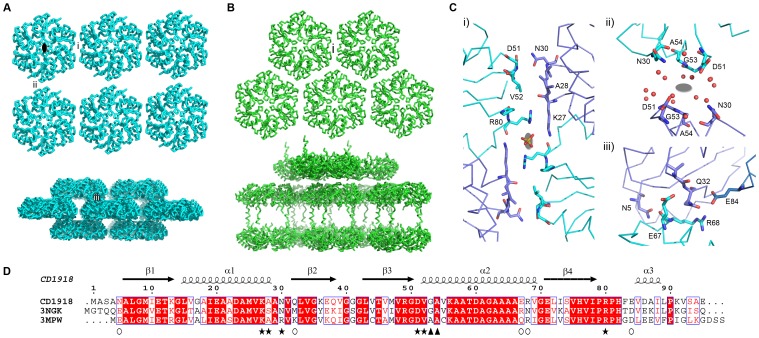
Crystal contacts of CD1918 and its homologues. **A**. Views of the crystal packing of CD1918 along the *a,c* (top) and *b,c* (bottom) planes with contact regions highlighted as roman numerals: *i*, *ii*, and *iii*. The crystallographic 2-fold generating the hexamer is shown as a black ellipse for a single multimer. **B**. crystal packing for EutM (PDBID: 3MPW), this protein packs into a regular two-dimensional lattice in the *a,c* plane (top) with a single conserved crystallographic interface, *i*. The *b,c* plane has widely spaced alternating layers distinct to CD1918, due to interactions between the C-terminal his-tag added to the construct for purification. **C**. Residues mediating CD1918 crystal contacts, panels *i*, *ii* and *iii* correspond to the interfaces marked in **A**. Interface *i* is conserved between CD1908 and its homologues, EutM and PduA, and in the crystal structures includes a coordinated sulphate ion; while interface *ii* is formed by solvent mediated contacts between chains. Interface *iii* is formed by non-conserved residues and forms a tight offset-layer packing between layers of hexamers. **D**. Sequence alignment of CD1918, PduA (PDBID: 3NGK) and EutM (PDBID: 3MPW). The secondary structure assignment for CD1918 is shown above the alignment. Conserved residues are shown in red, with strict conservation highlighted with a red background. Residues participating in crystal contacts shown in **C** are highlighted with *i*: stars; *ii:* triangles; *iii*: open circles.

Overexpression of CD1918 produced laminar features that appear rolled up in cross-section (Fig. 8C/D) that interfered with septation and cell division (Fig. 8D). These laminar features are roughly 10 nm in cross-section and of variable length. These structures are similar to those seen when both EtuA [49] and PduA are overexpressed [48] and to some degree correlate with the crystal packing of this protein and both PduA and EutM, which form 2D sheets and 10 nm thick filaments (Fig. 9A/B). The similarities seen in the crystal packing between these three proteins (Fig. 9C) and high level of sequence conservation seen in the regions that mediate these contacts (Fig. 9D) implies a conserved role for these proteins in the BMC shell. The His_6_ tag used to aid the purification of EutM clearly influences the packing of the layers (Fig. 9B), but the 2D arrays are identical to those seen for PduA, whose crystal packing is not influenced by its purification tag. It is not known whether these higher order structures require an additional protein, or small molecule co-factor to facilitate their formation *in vivo*. The structures seen for these proteins hint at the potential these proteins have for directing the assembly of the BMC shell.

## Discussion

*C. difficile* possesses an ethanolamine utilisation locus in its genome that encodes a two subunit ethanolamine ammonia lyase enzyme; accessory proteins required for the efficient utilisation of the carbon liberated by the breakdown of ethanolamine; six shell proteins, five with BMC protein domains and one with a carboxysome protein CcmL-like domain [50]; and proteins with putative regulatory functions. The requirements for the growth of *C. difficile* on ethanolamine as a nitrogen or carbon source have not yet been determined, but it is conceivable that the organism will grow on ethanolamine under fermentative conditions similar to those seen for *S. enterica,* or *E. faecalis* [15], [16], with the production of a functional BMC.

The sequestration of ethanolamine utilisation within a BMC is a widespread strategy among enteric pathogens [18] and shares a number of common features with the compartmentalisation of carbon fixation and the utilisation of propanediol by other bacterial species [26]. The shells of the BMCs must all act in the same way, to encapsulate enzymes and to provide a semi-permeable barrier between the cytosol and lumen of the compartment. Due to the shared function of the shell in different BMCs, the shell proteins display a high degree of structural conservation.

CD1908 is homologous to EutS of the *E. coli* ethanolamine utilisation microcompartment [34] and PduU of the propanediol utilisation microcompartment of *S. enterica* [40]. The role of these proteins in the microcompartment shell and possible function in substrate transport, or recruitment of enzymes to the lumen, is yet to be determined [51]. However, the common structures these proteins share, despite the different substrate specificities of the microcompartments they belong to, suggests a shared function independent of substrate. It has been suggested that there may be a degree of conformational flexibility in the β-barrel neck that allows it to act as a gated channel [40], in a similar manner to mechanosensitive ion channels [52]. The extension to the β-barrel seen in CD1908, which is shared with PduU but not EutS, may play a role in the recognition of substrates, or the recruitment of specific protein partners. If the protein does not act as a channel, it may have a common role in binding to and in organising enzymes within the BMC lumen. The role that CD1908 may play in the structure of the BMC shell is not clear; unlike EutS [37], which is able to form enclosed compartments when over-expressed alone in *E. coli*, CD1908 and its homologue PduU [48] form no higher order structures. Perhaps this difference in function may be a consequence of the flat hexameric oligomeric arrangement adopted by CD1908 and PduU, whereas EutS forms a bent hexamer. Why CD1908 should adopt a structure that is more closely related to PduU is unknown, but it hints at the plasticity and flexibility of these proteins and encourages further exploration of the functional role of these proteins with BMCs.

CD1918 is closely related to PduA [41] from *S. enterica,* EutM [42] from *E. coli* and EtuA from *C. kluyveri* [49]. The work of Parsons [48] and Heldt [49] indicates that these proteins are likely to play a central role in the organisation of microcompartment architecture. The sulphate ion that is found in its central pore implies a role in the transport of small polar, or charged molecules. The acetyl-phosphate produced by the phosphotransacetylase encoded by CD1920 and other EutD homologues is a possible candidate for its ligand.

The high-resolution structure of the EutQ family cupin, CD1925, highlights key differences between this class of β-barrel proteins and the metal binding cupins. In the absence of metal coordinating histidine residues a pocket is present that is lined with aromatic and hydrophobic residues. A pair of acidic residues (Glu100 and Asp102) lie within this pocket in an arrangement that is consistent with a role in ligand binding, or catalysis [53] (Fig. 5B/D) and identical to the arrangement found in the sugar epimerase cupins [45]. Isothermal titration calorimetry, co-crystallisation and soaking experiments were performed with substrates and co-factors from the ethanolamine utilisation pathway to determine the ligand for this protein. These experiments have thus far failed to identify a candidate ligand, or an activity for the protein. The negatively charged surface around the putative active site and the presence of a Glu/Asp pair within this cleft may point to a role in binding sugars, or a nucleotide, but this hypothesis will be the focus of future validation.

The localisation of EutQ proteins within cells expressing ethanolamine utilisation BMCs has not been determined, so it is not known whether the protein associates directly with the microcompartment, or is found within the cytosol. The presence of an unstructured N-terminal region is however consistent with the possibility that this protein is localised within the compartment [54]. The function of the protein as either a transporter, a partner in a signalling cascade, or an enzyme remains to be determined. Nevertheless, the amenability of this protein to high-resolution structural study will allow the mechanism of its action to be studied in detail when its physiological function is determined.

This work, and the body of literature that is available on the structures of microcompartment proteins and their interactions, indicates that there is some plasticity in the requirements for specific proteins to form the BMC shell. The comparison between the structure of CD1908 and its homologues highlights the importance of studying the same systems in different organisms to build a true picture of the function of BMCs. The atomic-level interactions between proteins within BMCs remain to be determined and will require structural studies of intact microcompartments. Knowledge of the relationships between the function and structure of BMCs is central to understanding the different substrate preferences and chemistries that they have and their role in the metabolism of pathogens, such as *C. difficile*.

## Materials and Methods

### Cloning, expression and purification

The open reading frames for the full length *C. difficile* ethanolamine utilisation (*eut*) locus proteins CD1908, CD1918 and CD1925 and the N-terminally truncated CD1925_(17–157)_ were amplified from genomic *C. difficile* 630 DNA template by PCR (see Table 3 for primer sequences) using the KOD DNA polymerase (Merck) with a standard protocol. The primer sequences were designed for insertion between NcoI and XhoI restriction sites in the pET28b vector (Invitrogen). N-terminal truncations of CD1925 were designed using sequence analysis to identify the putative start residue of the core fold and primers were made to produce a number of different internal start sites. The resulting PCR products were digested with the appropriate restriction endonucleases (Fermentas) according to the manufacturer's instructions and ligated into digested pET28b using T4 DNA ligase (Fermentas). Ligated plasmids were transformed into chemically competent *E. coli* Top10 cells and plasmids were isolated by miniprep (Fermentas). The expression plasmids generated were confirmed to match the published *C. difficile* 630 sequence (Genbank ID: NC_009089) by DNA sequencing (GATC Biotech).

**Table 3:** Primers.

Name	Sequence
CD1908F	CTC**CCATGG** *GCTTGACTGAAGAATCTAAGCAAAGAG*
CD1908R	CTC**CTCGAG** *TTATGTCCTTGTGATTTTTGTAGATGAGAAG*
CD1908R-His	CTC**CTCGAG** *TGTCCTTGTGATTTTTGTAGATGAGAAG*
CD1918F	CTC**CCATGG**GC*GCAAGTGCAAACGCATTA*
CD1918R	CTC**CTCGAG**TTACTCAGCTGATACCTTTGG
CD1918R-His	CTC**CTCGAG** *CTCAGCTGATACCTTTGG*
CD1925F	CTC**CCATGG**GC*GATATATCAAATATAGATAAAAAT*
CD1925R	CGC**CTCGAG** *TTAATTTTGAGATGCCCAATCTGCCGG*
CD1925_(17–157)_F	CTC**CCATGG**GC*CAAATAATAGAAGAAAAAATAAGT*
CD1925(_17–157)_R-His	CGC**CTCGAG** *ATTTTGAGATGCCCAATCTGCCGG*

Restriction sites are shown in bold face and genome complementary regions in italics.

All constructs were expressed in *E. coli* B834 (DE3) cells. A single colony was transferred to 200 ml of Zyp-5052 media [55] supplemented with 50 µg/ml kanamycin and grown at 310 K with shaking for 36 hours. Cells were harvested by centrifugation (4,000 *g*, 30 min) and washed with buffer A (50 mM Tris.HCl pH 8.0, 200 mM NaCl) before a second centrifugation step (4,000 *g*, 30 min) to pellet the cells.

Hexahistidine tagged proteins were purified as follows. Cells were resuspended in 40 ml of buffer HisA (50 mM Tris.HCl pH 8.0, 500 mM NaCl, 50 mM imidazole) and subjected to lysis by ultrasonication on ice. The lysate was clarified by centrifugation (35,000 *g*, 30 min) and the supernatant was filtered using a 0.45 µm syringe filter (Millipore). The filtered supernatant was loaded onto a 5 ml HisTrap column (GE Healthcare) equilibrated with buffer HisA and unbound protein was washed off with 5 column volumes of this buffer. The His_6_-tagged protein was eluted with buffer HisB (50 mM Tris.HCl pH 8.0, 500 mM NaCl, 500 mM Imidazole). The eluted protein was assessed for purity by SDS-PAGE, pooled and concentrated using a 10,000 kDa MWCO centrifugal concentrator (Amicon), prior to size exclusion chromatography using a Superdex S200 HR16/60 column (GE Healthcare) equilibrated with buffer A. Protein fractions were assessed by SDS-PAGE.

Untagged proteins were purified as follows. Cells were resuspended in 40 ml of buffer QA (50 mM Tris.HCl pH 8.0) and subjected to lysis by ultrasonication on ice. The lysate was clarified by centrifugation (35,000 *g*, 30 min) and the supernatant was filtered using a 0.45 µm syringe filter (Millipore). The filtered supernatant was loaded onto a 5 ml Q-sepharose column (GE Healthcare) equilibrated with buffer QA. Unbound sample was washed off with 10 column volumes of buffer QA and protein was eluted with a linear gradient of 0–100% buffer QB (50 mM Tris.HCl pH 8.0, 1 M NaCl) over 25 column volumes. Peak fractions were analysed by SDS-PAGE and fractions containing the protein of interest were pooled and subjected to size exclusion chromatography as described above.

### Crystallisation and data collection

Purified hexa-histidine tagged CD1908 (CD1908-His) was concentrated to 10 mg/ml in Milli-Q H_2_O and crystallised by sitting drop vapour diffusion in drops of 100 nl protein plus 100 nl crystallisation solution, over 100 µl of the latter. Crystals were obtained in 24% (w/v) PEG 1500, 20% (v/v) glycerol; these were harvested from the well using a CryoLoop (Hampton Research) and flash cooled directly in liquid nitrogen. CD1918-His was concentrated to 10 mg/ml in Milli-Q H_2_O and crystallised as described for CD1908 in drops supplemented with crystallisation solution containing 200 mM Li_2_SO_4_, 100 mM phosphate/citrate buffer pH 4.2, 20% (w/v) PEG 1000. Crystals were harvested through paratone oil using a CryoLoop and flash cooled in liquid nitrogen. CD1925_(17–157)_-His was concentrated to 8 mg/ml in buffer A and crystallised by hanging drop vapour diffusion in drops of 1 µl protein plus 1 µl crystallisation solution over 1 ml of the latter. Crystals were obtained in 100 mM sodium acetate pH 4.5, 35% (w/v) PEG 6000, 200 mM MgCl_2_, these were harvested by transfer to a cryoprotection solution containing the well solution supplemented with 20% v/v PEG300 and flash cooled in liquid nitrogen. All crystallographic data were collected on beamlines I02 and I04 at Diamond Light Source (Didcot, UK) at 100 K using ADSC CCD detectors. Diffraction data were integrated using iMosflm [56] or XDS [57] and scaled and merged with Scala [58]. Data collection and refinement statistics are shown in Table 2.

### Structure solution and analysis

All structures were solved by molecular replacement using Phaser [59], molecular replacement models used are shown in Table 2. Refinement of the coordinates, TLS parameters and atomic temperature factors (anisotropic in the case of CD1908 and CD1925_(17–152)_) was carried out using Phenix.refine [60]. Model building was performed using Coot [61]. The secondary structure and stereochemistry of the models was analysed by MolProbity [62]. Sequence alignment was performed using ClustalW [63] and the corresponding figures were generated using ESPript [64]. Oligomerisation states and values of buried surface areas were calculated using the PISA server [65]. Structural superimpositions were calculated using Coot. Crystallographic figures were generated with PyMOL [66].

### Thin-section transmission electron microscopy

*E. coli* B834 cells transformed with the plasmids for untagged CD1908, CD1918 and CD1925 were grown to mid-log phase in Luria-Bertani media supplemented with 50 µg/ml kanamycin at 310 K and induced with 1 mM final concentration of IPTG and harvested after 3 hours. 1 ml of these cells and un-induced controls were fixed in 2.5% (v/v) glutaraldehyde, 50 mM sodium cacodylate pH 7.0 for 24 hours at 4°C. Cells were subsequently immobilized in 2% (w/v) water-agar and post-fixed in 1.5% (w/v) osmium tetroxide in 50 mM sodium cacodylate pH 7.0 for 1 hour at 4°C followed by dehydration in an ethanol series. The final 70% dehydration step was supplemented with 1% (w/v) uranyl acetate and was performed overnight at 21°C. 100 nm sections were post-stained with 2% (w/v) uranyl acetate and analysed using a Philips CM100 transmission electron microscope.

### Accession Codes

Refined coordinates and structure factors have been deposited at the PDB with the following accession numbers: CD1908 PDB ID: 4AXI; CD1918 PDB ID: 4AXJ; CD1925 PDB ID: 4AXO.

## Supporting Information

Supporting Information S1 Organisation of C. difficile ethanolamine utilisation operon.
(DOCX)
