# Supplementary material for: Structural Insight into the Clostridium difficile Ethanolamine Utilisation Microcompartment
Source: PLoS One. 2012 Oct 29;7(10):e48360. doi: 10.1371/journal.pone.0048360 (PMC3483176; doi:10.1371/journal.pone.0048360)
Supplement: Supporting Information S1 — Organisation of C. difficile ethanolamine utilisation operon. (DOCX) [file pone.0048360.s001.docx]

Supporting Information

Organisation of *C. difficile* ethanolamine utilisation operon

Enzymes for ethanolamine breakdown and utilisation

The *C. difficile* *eut* operon has twenty annotated genes (Fig. 1, Table 1), including the enzymes required for ethanolamine breakdown and utilisation. These enzymes include the two-subunit ethanolamine ammonia lyase, *eutB* and *eutC* (*CD1913-14*) [[1](#_ENREF_1),[2](#_ENREF_2)]; a cyano-cobalamin reactivating enzyme, *eutA* (*CD1912*), and a cobalamin adenosyl transferase, *eutT* (*CD1919*) [[3](#_ENREF_3)]. Genes encoding enzymes involved in the conversion of acetaldehyde into acetyl-coA, *eutE* (*CD1917*); or ethanol, *eutG* (*CD1907*), are also present [[4](#_ENREF_4)]. The operon also contains a gene encoding a phosphotransacetylase, *eutD* (*CD1920*), which produces acetyl-phosphate from the acetyl-coA produced by the *CD1917* gene product. The acetyl phosphate is then available for substrate level phosphorylation of ADP to ATP by a cytoplasmic acetate kinase [[5](#_ENREF_5),[6](#_ENREF_6)]. The proteins encoded by *CD1909* and *CD1925* have no experimentally determined function [[7](#_ENREF_7)]. The *CD1909 gene* product is homologous to EutP, which has a P-loop GTP binding motif and is the proposed reactivating enzyme for the AdoCbl cofactor [[8](#_ENREF_8)]. *CD1925* encodes a conserved protein that belongs to the EutQ family of cupin barrels, although its role in ethanolamine utilisation is currently unknown; deletion of this gene in *Salmonella enterica* has no growth phenotype [[9](#_ENREF_9)]. A trans-membrane permease for uncharged ethanolamine transporter, EutH, is encoded by *CD1924* [[10](#_ENREF_10)].

**Bacterial microcompartment shell**

The operon has six genes that encode proteins with homology to the carboxysome shell proteins: *CD1908*, *CD1915*, *CD1916*, *CD1918*, *CD1922*, and *CD1923*. *CD1908* and *CD1918* encode proteins with a single conserved BMC domain; *CD1915* and *CD1923* encode proteins with tandem conserved domains, the latter having homology to the iron sulphur containing PduT protein [[11](#_ENREF_11)]. *CD1916* encodes a protein with a single BMC domain and a C-terminal domain with no significant sequence homology to any domain of known function and which is found only in the *C. difficile* genome sequence. *CD1922* encodes a protein with homology to the carboxysome protein CcmL which is thought to form the pentameric vertices of the enclosed compartment [[12](#_ENREF_12)].

**Regulation of the operon**

Numerous strategies exist to control the transcription and translation of genes encoded within the *eut* operon. For example, *S. enterica* possesses an AraC-type transcriptional activator that requires the presence of both AdoCbl and ethanolamine for activity [[13](#_ENREF_13)], while *E. faecalis* has a two-component regulator that senses ethanolamine and an AdoCbl dependent riboswitch that modulates transcription [[14](#_ENREF_14)]. The *C. difficile* *eut* operon has two strong consensus σ^A^ promoter elements, one upstream of *CD1907* and the second upstream of *CD1908* (Figure 1). Genes encoding a two-component system, comprising the proposed ethanolamine-sensing sensor histidine kinase EutW, and its cognate response regulator EutV are present (*CD1910-11*); it has been proposed that the activated response regulator binds to two ANTAR recognition motifs found in the intergenic regions upstream of *CD1908* and *CD1912* [[15](#_ENREF_15),[16](#_ENREF_16)]. The presence of this two-component system implies that the expression of the *eut* locus is induced in response to changes in intracellular ethanolamine levels. There is also an open reading frame on the complementary strand at the end of the operon (*CD1926*), with homology to the AraC type DNA binding domain of the EutR transcriptional regulator [[13](#_ENREF_13),[17](#_ENREF_17)]. The DNA binding specificity of the protein encoded by this gene has not been experimentally determined. A comparative analysis of bacterial genomes with *eut* operons has identified a number of potential DNA consensus sequences for EutR binding [[8](#_ENREF_8)], although analysis of the *C. difficile eut* operon does not identify any regions showing these consensus sequences. Given the dearth of information on this protein, and the fact that the protein encoded by the *CD1926* possesses only the DNA binding domain, it is not clear if it is functional, what ligand sensing properties it may still possess, and if it regulates this operon in *C. difficile.*

**Supporting References**

1. Bradbeer C (1965) The clostridial fermentations of choline and ethanolamine. II. Requirement for a cobamide coenzyme by an ethanolamine deaminase. J Biol Chem 240: 4675-4681.

2. Bradbeer C (1965) The clostridial fermentations of choline and ethanolamine. 1. Preparation and properties of cell-free extracts. J Biol Chem 240: 4669-4674.

3. Buan NR, Suh SJ, Escalante-Semerena JC (2004) The eutT gene of Salmonella enterica Encodes an oxygen-labile, metal-containing ATP:corrinoid adenosyltransferase enzyme. J Bacteriol 186: 5708-5714.

4. Stojiljkovic I, Baumler AJ, Heffron F (1995) Ethanolamine utilization in Salmonella typhimurium: nucleotide sequence, protein expression, and mutational analysis of the cchA cchB eutE eutJ eutG eutH gene cluster. J Bacteriol 177: 1357-1366.

5. Starai VJ, Garrity J, Escalante-Semerena JC (2005) Acetate excretion during growth of Salmonella enterica on ethanolamine requires phosphotransacetylase (EutD) activity, and acetate recapture requires acetyl-CoA synthetase (Acs) and phosphotransacetylase (Pta) activities. Microbiology 151: 3793-3801.

6. Brinsmade SR, Escalante-Semerena JC (2004) The eutD gene of Salmonella enterica encodes a protein with phosphotransacetylase enzyme activity. J Bacteriol 186: 1890-1892.

7. Brinsmade SR, Paldon T, Escalante-Semerena JC (2005) Minimal functions and physiological conditions required for growth of salmonella enterica on ethanolamine in the absence of the metabolosome. J Bacteriol 187: 8039-8046.

8. Tsoy O, Ravcheev D, Mushegian A (2009) Comparative genomics of ethanolamine utilization. J Bacteriol 191: 7157-7164.

9. Kofoid E, Rappleye C, Stojiljkovic I, Roth J (1999) The 17-gene ethanolamine (eut) operon of Salmonella typhimurium encodes five homologues of carboxysome shell proteins. J Bacteriol 181: 5317-5329.

10. Penrod JT, Mace CC, Roth JR (2004) A pH-sensitive function and phenotype: evidence that EutH facilitates diffusion of uncharged ethanolamine in Salmonella enterica. J Bacteriol 186: 6885-6890.

11. Pang A, Warren MJ, Pickersgill RW (2011) Structure of PduT, a trimeric bacterial microcompartment protein with a 4Fe-4S cluster-binding site. Acta Crystallogr D Biol Crystallogr 67: 91-96.

12. Tanaka S, Kerfeld CA, Sawaya MR, Cai F, Heinhorst S, et al. (2008) Atomic-level models of the bacterial carboxysome shell. Science 319: 1083-1086.

13. Roof DM, Roth JR (1992) Autogenous regulation of ethanolamine utilization by a transcriptional activator of the eut operon in Salmonella typhimurium. J Bacteriol 174: 6634-6643.

14. Baker KA, Perego M (2011) Transcription antitermination by a phosphorylated response regulator and cobalamin-dependent termination at a B riboswitch contribute to ethanolamine utilization in Enterococcus faecalis. J Bacteriol 193: 2575-2586.

15. Fox KA, Ramesh A, Stearns JE, Bourgogne A, Reyes-Jara A, et al. (2009) Multiple posttranscriptional regulatory mechanisms partner to control ethanolamine utilization in Enterococcus faecalis. Proc Natl Acad Sci U S A 106: 4435-4440.

16. Ramesh A, Debroy S, Goodson JR, Fox KA, Faz H, et al. (2012) The Mechanism for RNA Recognition by ANTAR Regulators of Gene Expression. PLoS Genet 8: e1002666.

17. Roof DM, Roth JR (1989) Functions required for vitamin B12-dependent ethanolamine utilization in Salmonella typhimurium. J Bacteriol 171: 3316-3323.
